# Expression of the epithelial-mesenchymal transition-related proteins and their clinical significance in lung adenocarcinoma

**DOI:** 10.1186/1746-1596-8-89

**Published:** 2013-05-24

**Authors:** Yongli Shi, Hongyan Wu, Mingyi Zhang, Lei Ding, Fanqing Meng, Xiangshan Fan

**Affiliations:** 1 Department of Pathology, Nanjing Drum Tower Hospital, the Affiliated Hospital of Nanjing University Medical School, Nanjing, China; 2 Department of Pathology, Huaian No.1 People’s Hospital, Huaian, China; 3 Department of Huaiyin Hospital, Huaian, China

**Keywords:** Epithelial–mesenchymal transition, Lung adenocarcinoma, Survival analysis, Tissue array analysis

## Abstract

**Background:**

Epithelial-mesenchymal transition (EMT) is defined as switching of polarized epithelial cells to a migratory fibroblastoid phenotype. EMT is known to be involved in the progression and metastasis of various cancers. The aim was to evaluate that whether EMT-related proteins' alterations are associated with clinicopathological features and prognosis in lung adenocarcinoma.

**Methods:**

The expression of EMT-related proteins including cytokeratin, E-cadherin, TTF-1, β-catenin, vimentin, Snail, Twist, CD44 was evaluated by immunohistochemistry using a tissue array method in the lung adenocarcinoma tissues of 95 patients. In addition, clinicopathological characteristics and survival were compared with the expression of EMT-related proteins.

**Results:**

Loss of epithelial proteins and/or acquisition of the expression of mesenchymal proteins were observed in lung adenocarcinoma. These proteins’ alteration was associated with poor cell differentiation and poor patients’ outcome, respectively. Subjects were divided into two groups according to the number of EMT-related proteins’ alteration. A higher number of EMT-related proteins’ alteration was found to be significantly associated with unfavorable outcome. Multivariate analysis showed that a higher number of EMT-related proteins’ alteration was independently associated with poor prognosis.

**Conclusions:**

The number of EMT-related proteins’ alteration is a significant prognostic marker to predict overall survival in patients with lung adenocarcinoma. The information generated will be valuable for the prognosis of patients with lung adenocarcinoma.

**Virtual slides:**

The virtual slides for this article can be found here: http://www.diagnosticpathology.diagnomx.eu/vs/1007838329872974

## Background

Non-small cell lung cancer is the leading cause of cancer death worldwide. Among non–small cell lung cancer variants, adenocarcinoma is the most common histological subtype. Surgical resection is the treatment of choice for early-stage adenocarcinoma. However, tumor recurrence and metastasis are the most common events encountered after resection that lead to mortality [1,2]. Chemotherapy and radiotherapy are common treatment modalities applied to patients with recurrent adenocarcinoma [3], but the combination modality did not significantly improve patients’ survival. Since tumor metastasis is the main obstacle for long-term survival after surgical resection, identification of molecular markers related to metastasis may better predict the prognosis in patients with lung adenocarcinoma.

Epithelial-mesenchymal transition (EMT) consists of a rapid and often reversible change of cell phenotype. During EMT, cells lose or redistribute epithelial proteins and acquire mesenchymal proteins. As a result, cells loss epithelial polarity and acquire a spindle-shaped, highly motile fibroblastoid phenotype. This transition involves which confer upon cells the ability to pass through the basement membrane [4-7] and a developmental program of tumor cells [8,9]. The phenomenon of EMT is proved during various of numerous cancers, e.g. pancreatic cancer, gastric, and colorectal carcinomas [10-13]. It is an important event in the progression, invasion and metastasis of carcinomas which have a particularly dismal prognosis [5,7,14].

In this study, using a tissue array method, we investigated the expression of known EMT-related proteins including cytokeratin, E-cadherin, TTF-1, β-catenin, vimentin, Snail, Twist, CD44 in lung adenocarcinoma patients’ samples. The aim was to evaluate changes in EMT-related protein and to investigate their association with clinicopathological parameters and prognosis in lung adenocarcinoma.

## Materials and methods

### Patients’ samples

From January 2007 to December 2009, 95 patients undergoing surgical resection for lung adenocarcinoma at Nanjing Drum Tower Hospital were enrolled in this study. Clinicopathological parameters such as age, gender, cell differentiation and pathological stage were evaluated by reviewing pathological records. The mean patient age was 58 years. Samples were obtained from 56(58.8%) male and 39 (41.2%) female patients, and there were 51 (53.7%, I-II stage) cases of early-stage adenocarcinoma and 44 (46.3%, III-IV stage) cases of advanced-stage adenocarcinoma. Thirty-three patients (34.7%) were poorly differentiated adenocarcinoma and sixty-two (65.3%) were well and moderately differentiated adenocarcinoma. Outcomes were determined from the date of surgery until death or June 2012. The study was approved by the Medical Ethics Committee of the Affiliated Drum Tower Hospital of Nanjing University Medical School.

### Tissue microarray construction

The tissue microarray was created from tissue blocks that had been stored at approximately 24°C. Hematoxylin and eosin-stained sections were reviewed to select representative areas of tumor, and then to acquire cores for the microarray. The tissue microarray block was constructed with a precision instrument (Beecher Instruments, Sun Prairie, WI). Core tissue biopsies (1 mm in diameter) were taken from individual donor blocks and arranged in a new recipient beeswax block (tissue array block). Sections (2 μm) were consecutively cut and placed on positively charged slides for use in immunohistochemical staining.

### Immunohistochemical staining, scoring

Immunohistochemistry for cytokeratin, E-cadherin, TTF-1, β-catenin, vimentin, Snail, Twist, CD44 were performed (Table 1). Slides were placed in a 60°C oven for 30 minutes, deparaffinized, and rehydrated through xylenes and graded ethanol solutions to water. Antigen retrieval was performed by a steamer method in which the specimens were placed in a 0.01 mol/L EDTA solution (pH = 8) for 30 minutes at 94°C using a steamer. Primary antibodies were applied overnight at 4°C, followed by incubation at room temperature with horseradish peroxidase (HRP) conjugated anti-mouse secondary antibody for cases of each primary antibody. Appropriate positive and negative controls were used for the immunohistochemical analysis. After staining, if any case on one slide represented a missing core missing, displacement, or undetectable cancer cells or mucosal glands, the corresponding paraffin-embedded tissue block was recut and restained. Evaluation of the cell staining reaction was performed in accordance with the following immunoreactive score (IRS) proposed by Remmele and Stegner with slight modification as follows: IRS = SI (staining intensity) × PP (percentage of positive cells). SI was determined as 0, negative; 1, weak; 2, moderate; and 3, strong. PP was defined as 0, negative; 1, 1-10% positive cells; 2, 11-50% positive cells; 3, 51-80% positive cells; 4, *>*80% positive cells. IRS value ≥ 4 was considered as a positive staining result [15].

**Table 1:** Antibodies used in the immunohistochemical study

**Antibody**	**Source**	**Non-neoplastic mucosa**	**Altered expression in cancer**
Cytokeratin	mouse monoclonal, clone AE1/AE3, 1:200, Dako	Cytoplasmic	Loss
E-cadherin	mouse monoclonal, clone HECD-1, 1:200, Zymed	Membranous	Loss
TTF-1	mouse monoclonal, clone 8G7G3/1, 1:200, Dako	Nuclear	Loss
β-catenin	mouse monoclonal, clone CAT-5H10, 1:500, Zymed	Membranous	Cytoplasmic or Nuclea
Vimentin	mouse monoclonal, clone vim-3B4, 1:400, Dako	Negative	Cytoplasmic
Snail	rabbit polyclonal, ab 85931, 1:500, Abcam	Negative	Nuclear
Twist	rabbit polyclonal, ab 50581, 1:500, Abcam	Negative	Cytoplasmic
CD44	mouse monoclonal, clone DF1485, 1:250, Dako	Negative	Membranous

### Statistical analysis

Comparison of the variables was performed using Student’s t test, Fisher’s exact test or Pearson’s χ2 test, depending on the nature of the data. Correlation analysis was performed using Spearman’s correlation analysis. Survival curves were estimated using the Kaplan–Meier product-limit method, and the significance of differences between survival curves was determined using the log rank test. Multivariate analysis was performed by Cox proportional hazards regression modeling. All statistical tests were two-sided, and statistical significance was accepted at the *p* < 0.05 level. All analyses were performed using SPSS version 11.0.

## Results

### The alteration of EMT-related proteins and their correlation with clinicopathological factors in lung adenocarcinoma

**Figure 1:**
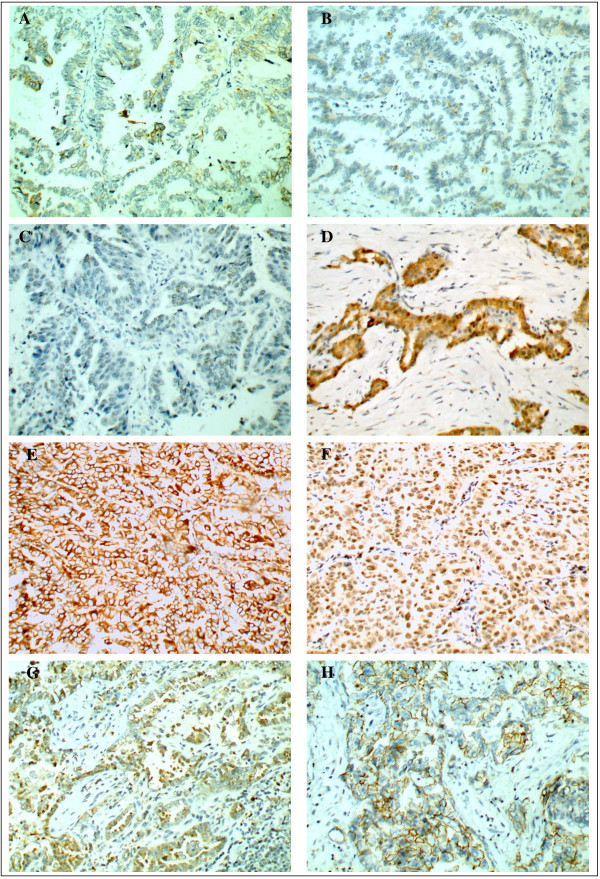
**Immunohistochemistry findings showing epithelial protein loss (A–C) and mesenchymal protein expression (D-H) in lung adenocarcinoma. A** Cytoplasmic cytokeratin loss. **B** Membranous E-cadherin loss. **C** Nuclear TTF-1 loss. **D** Nuclear and Cytoplasmic β-catenin expression. **E** Cytoplasmic vimentin expression. **F** Nuclear Snail expression. **G** Cytoplasmic Twist expression. **H** Membranous CD44 expression.

Losing frequencies of Epithelial proteins were 25.3% for cytokeratin, 56.8% for E-cadherin, 38.9% for TTF-1. Aberrant expression frequencies of mesenchymal proteins were 47.4% for β-catenin, 40.0% for vimentin, 60.0%for Snail, 68.4% for Twist, 41.1% for CD44 (Figure 1).

The alteration of TTF-1, vimentin, Snail and CD44 were found to be significantly associated with poorly cell differentiation. The alteration of vimentin was found to be significantly associated with higher TNM stage. No differentiation existed in tumor EMT-related proteins expression among groups of age, gender. The association of EMT-related proteins' staining and the clinicopathological characteristics are summarized in Table 2.

**Table 2:** Association between EMT-related proteins and clinicopathological variables

	**Cell differentiation**		***p*-value**	**TNM stage**		***p*-value**
	**Poorly**	**Well and moderately**		**I-II**	**III-IV**	
	**(%)**	**(%)**		**(%)**	**(%)**	
Loss of epithelial protein expression						
Cytokeratin	8(24.2)	16(25.8)	0.867	11(21.6)	13(29.5)	0.372
E-cadherin	19(57.6)	35(56.5)	0.916	29(56.9)	25(56.8)	0.997
TTF-1	20(60.6)	17(27.4)	0.002	16(31.4)	21(47.4)	0.103
Acquisition of mesenchymal protein expression						
β-catenin	19(57.6)	26(41.9)	0.146	23(45.1)	22(50.0)	0.633
Vimentin	18(54.5)	20(32.3)	0.035	14(27.5)	24(54.5)	0.007
Snail	26(78.7)	31(50.0)	0.006	29(56.9)	28(63.6)	0.502
Twist	25(75.8)	40(64.5)	0.262	35(68.6)	30(68.2)	0.963
CD44	20(60.6)	19(30.6)	0.005	22(43.1)	17(38.6)	0.657

The association between epithelial protein and mesenchymal protein expression was demonstrated by the Pearson χ2 test. Losing of cytokeratin and E-cadherin were significantly associated with β-catenin (*p* = 0.029, *p* < 0.001) and Twist (*p* = 0.006, *p* = 0.028) expression. Losing of TTF-1 was found to be significantly associated with β-catenin (*p* = 0.006) and vimentin (*p* = 0.026) expression.

### The alteration of EMT-related proteins as prognostic factors in patients with lung adenocarcinoma

To investigate the prognostic impact of EMT-related proteins' alterations in lung adenocarcinoma, Kaplan-Meier survival analyses were carried out. In terms of the epithelial proteins, the loss of cytokeratin (*p* = 0.0224), E-cadherin (*p* = 0.0132) and TTF-1 (*p* = 0.0208) were found to be significantly associated with a poor outcome. For mesenchymal proteins, the expression of vimentin (*p* = 0.0022), Snail (*p* = 0.0454), CD44 (*p* = 0.0167) were found to be significantly associated with a poor outcome (Table 3).

**Table 3:** Univariate analysis for overall survivals of 95 patients with lung adenocarcinoma

**Variable**	**n**	**Log-rank**	***p-*value**
Cytokeratin	+71	5.22	0.0224
	-24		
E-cadherin	+41	6.14	0.0132
	-54		
TTF-1	+58	5.34	0.0208
	-37		
β-catenin	+45	0.19	0.6612
	-50		
Vimentin	+38	9.38	0.0022
	-57			
Snail	+57	4.00	0.0454	
	-38			
Twist	+65	1.35	0.2458	
	-30			
CD44	+39	5.73	0.0167	
	-56			

**Figure 2:**
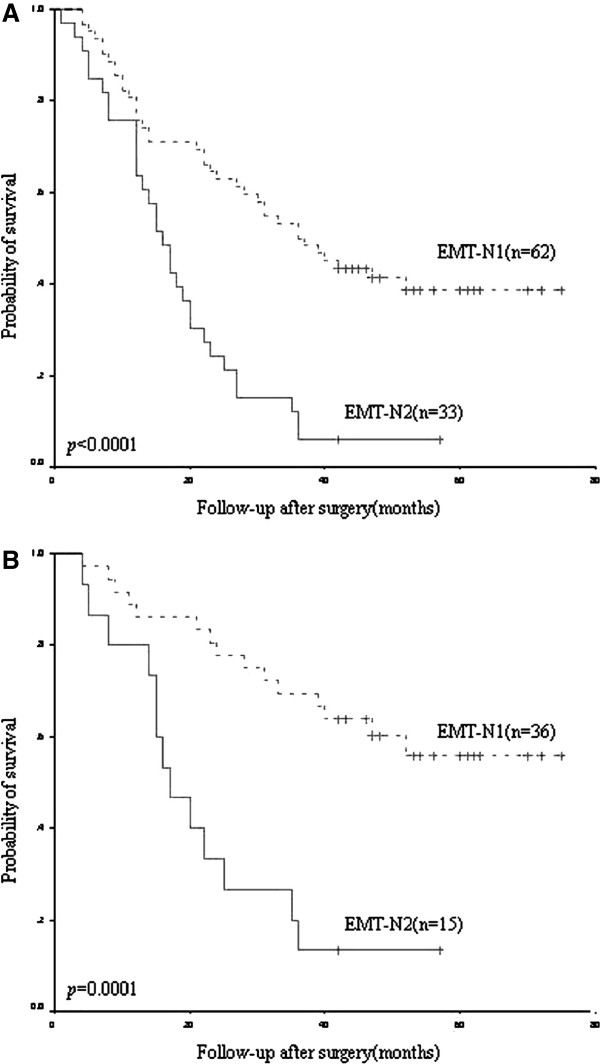
**Survival curves using the Kaplan–Meier method by log rank test. A** EMT-N in all cases. **B** EMT-N in TNMI-II cases.

To investigate the accumulative effects of EMT-related proteins’ alteration on the prognosis of lung adenocarcinoma, the patients were divided into two groups according to the number of EMT-related proteins’ alteration: EMT-N1 (one to four protein changes occurred in 62 cases), EMT-N2 (more than four protein changes occurred in 33 cases). Patients with EMT-N2 showed a more unfavorable prognosis than those with EMT-N1 (*p* < 0.0001, Figure 2A). TNM stage and EMT-N were entered into multivariate analyses. To control for potential confounders, age, gender, and cell differentiation were also entered into multivariate analyses. Multivariate analyses showed that TNM stage (*p* < 0.001), cell differentiation (*p* = 0.030) and EMT-N (*p* = 0.002) were independent prognostic markers for overall survival (Table 4).

**Table 4:** Multivariate analyses for overall survivals of 95 patients with lung adenocarcinoma

**Variables**		**HR(95% CI)**	***p*-value**
TNM stage	I-II	0.399(0.241 to 0.659)	<0.001
	III-IV		
Cell differentiation	Poorly	1.772(1.056 to 2.974)	0.030
	Well and moderately		
TNM-N	TNM-N1	0.430(0.255 to 0.726)	0.002
	TNM-N2		

Patients with EMT-N2 showed a more unfavorable prognosis than those with EMT-N1, and this was true for early-stage lung adenocarcinoma (stage I and II, n = 51). For early-stage lung adenocarcinoma in the study, patients with EMT-N2 had significantly worse overall survival than those with EMT-N1 (*p* = 0.0001, Figure 2B). Multivariate analysis identified EMT-N was also an independent prognostic marker for overall survival in early-stage lung adenocarcinoma (HR = 0.405, 95% CI = 0.180 to 0.908, *p* = 0.028).

## Discussion

Non-small cell lung cancer (NSCLC) is the most predominant type of lung cancer and the leading cause of cancer death worldwide [16]. Deletions or insertions in exon 19 and point mutations in exons 18 and 21 in the epidermal growth factor receptor are special diagnostic value in advanced-stage non-small cell lung cancer patients [17,18]. Expression of oncofetal protein IMP3 correlates with distant metastases regardless of histological subtype of lung adenocarcinoma [19]. Promoter methylation was associated with clinicopathologic characteristics and may be served as a potentially increased risk factor for pleural indentation of NSCLC [20]. These groups have used different sets of markers to predict the prognosis and survival of patients with NSCLC with some success. However, there is little by using a combination of metastasis-related markers. Epithelial-mesenchymal transition (EMT) is considered to be one of the major molecular mechanisms inducing tumour invasion and metastasis. Some study showed that centrally located tumour cells stained positively for epithelial markers, but it was absent at the invasive front of the tumour in lung cancer [21-23]. So the expression of EMT-related proteins which related to metastasis may better reflect and predict the prognosis and survival in patients with NSCLC. In our study the aim was to evaluate changes in EMT-related proteins and to investigate their association with prognosis in lung adenocarcinoma.

As the epithelial markers, E-cadherin and cytokeratin expression is strongly related to positive serosal involvement, infiltrating type, poorly differentiated histology [13,24]. Thyroid transcription factor-1 (TTF-1) is a transcription factor that is expressed in approximately 75% of lung adenocarcinoma. Several studies demonstrated an independent lower risk of death for lung adenocarcinoma patients whose tumor expresses positive TTF1 staining [25]. Recently one paper suggested that TTF-1 is an important EMT-related marker. It inhibits EMT and restores epithelial phenotype in lung adenocarcinoma cells [26]. As a result, a novel aspect of TTF-1 is that losing expression of TTF-1 made tumor cells generating EMT and then resulting in the worse prognosis of patients. The above reports and our findings indicate that loss of E-cadherin, cytokeratin and TTF1 expression is an important indicator of EMT. The regulators involving mesenchumal differentiation such as β-catenin, Snail, Twist are considered to regulate EMT by strong repression of E-cadherin expression [11,27,28]. Our study showed that the acquisition of mesenchymal protein expression tended to correlate with loss of epithelial protein expression. CD44 has been identified as a specific marker of cancer stem cells. In addition, CD44 plays an important role in tumor cells undergoing an EMT-like process and associated with cancer progression [29,30]. In the present study we have demonstrated that the expression of various EMT-related proteins is associated with a poor prognosis in lung adenocarcinoma. Our results support previous reports where the expression of various EMT-related molecule were associated with neoplastic progression and poor survival in some malignancies.

Characteristics of EMT include complete loss of epithelial polarity, loss of epithelial markers and acquisition of mesenchymal markers. In addition to processes involving complete EMT, many processes occurring during development and in adult organisms involve only a transient loss of epithelial polarity without full acquisition of mesenchymal characteristics. We have defined as partial EMT [31,32]. Partial EMT has been suggested to occur in some metastatic cancers and the number of EMT-related proteins’ alteration may reflect the degree of EMT in a way [33]. So in our study, we divided the patients into two groups according to the number of EMT-related proteins’ alteration. The results showed that the patients with higher number of EMT-related proteins’ alteration had a significantly shorter overall survival. In multivariate analyses the number of EMT-related proteins’ alteration was significant independent prognostic indicators for overall survivals. This finding suggests that higher number of EMT-related proteins’ alteration may be significantly related to tumor progression and metastasis.

There is increasing evidence to support the role of postoperative adjuvant chemotherapy in locally advanced-stage lung cancer. However, the effect of adjuvant chemotherapy in early-stage adenocarcinoma remains to be determined [34]. In the study we analyzed the predictive ability of the number of EMT-related proteins’ alteration in patients with early-stage lung adenocarcinoma. For early-stage lung adenocarcinoma (stage I and II) there is a trend toward worse overall survival in patients with a higher number of EMT-related proteins’ alteration. The number of EMT-related proteins’ alteration was significant independent prognostic indicators for overall survivals for early-stage adenocarcinoma in multivariate analyses. The results suggest that the number of EMT-related proteins’ alteration may be able to identify the poor prognostic cases in early-stage lung adenocarcinoma. Adjuvant therapy may be considered in patients with early-stage lung adenocarcinoma when patients have a higher number of EMT-related proteins’ alteration.

## Conclusions

The present study showed mainly two results. One was that the number of EMT-related proteins’ alteration is a independent prognostic marker to predict overall survival in patients with lung adenocarcinoma. The information generated will be valuable for the prognosis of patients with lung adenocarcinoma. The other was that the number of EMT-related proteins’ alteration is also a independent prognostic marker to predict overall in patients with early-stage (stage I and II) lung adenocarcinoma. The information generated will be valuable for determining the adjuvant chemotherapy for patients with early-stage lung adenocarcinoma.

## Consent

Written informed consent was obtained from the patient for publication of this report and any accompanying images.

## Competing interests

The authors declare that they have no competing interests.

## Authors’ contributions

FQM, YLS and XSF designed the study, analysed histological slides and wrote the manuscript. LD, HYW and MYZ collected the patients’ clinical information and obtained the follow-up data. All authors have read and approved the final manuscript.
